# Moroccan Children With Helicobacter pylori Infection: Demographics, Clinical Features, and Histological Findings

**DOI:** 10.7759/cureus.40740

**Published:** 2023-06-21

**Authors:** Lahbib Hibaoui, Abdelhamid Massik, Ghita Yahyaoui, Mustapha Mahmoud, Naoual Hmass, Laila Chbani, Mounia Lakhdar Idrissi, Moustapha Hida

**Affiliations:** 1 Biomedical and Translational Research Laboratory, Faculty of Medicine and Pharmacy, Sidi Mohamed Ben Abdellah University, Fez, MAR; 2 Department of Microbiology and Molecular Biology, Hassan II University Hospital, Fez, MAR; 3 Department of Surgical Pathology, Hassan II University Hospital, Fez, MAR; 4 Laboratory of Epidemiology, Clinical Research and Community Medicine, Faculty of Medicine and Pharmacy, Sidi Mohamed Ben Abdellah University, Fez, MAR; 5 Department of Paediatrics, Hassan II University Hospital, Fez, MAR

**Keywords:** histological characteristics, clinical features, gastritis, children, helicobacter pylori infection

## Abstract

Background: Infesting nearly half of the world's population, *Helicobacter pylori* is thought to cause peptic ulcers and gastric adenocarcinoma. Several studies have examined the association between *H. pylori* and socioeconomic, clinical, and histological factors in pediatric populations. Similarly, this study aimed to describe the characteristics of *H. pylori* infection in Moroccan children.

Methods: Patients aged 1-17 years who underwent upper gastrointestinal endoscopy over a period of two years from January 2019 to January 2021 were included in this study. Gastric biopsies from the antrum and corpus of the stomach were collected. Detection of *H. pylori* infection was confirmed by Giemsa stain. Demographic data and clinical and endoscopic characteristics were collected and histopathological findings with gastritis scoring were recorded according to the Sydney System.

Results: In 213 children, 95 (45%) were found to be infected with *H. pylori*, and the infection rates increased as the children aged. While no significant relationship between the infection of *H. pylori* and all symptoms was founded, a significant association was found in nodular gastritis (p<0.05), and 98% of the infected children had chronic inflammation, which was active in 22% and atrophic in 47%. The atrophy and activity were absent or mild, and the inflammation was mild to moderate.

Conclusion: According to this study, nodular gastritis and nonspecific symptoms were related to *H. pylori* infection in Moroccan children. In addition, the association between this disease and gastric atrophy in our study needs the monitoring of the mucosa of Moroccan children with gastritis and identifying factors that may contribute to gastric cancer.

## Introduction

*Helicobacter pylori* is one of the most prevalent human pathogens and the key pathogenic bacterium in pediatric gastroenterology. Affecting more than half of the world's population, this bacterium continues to be a major public health issue [1]. It is a major cause of chronic gastritis, and peptic ulcer diseases, and a significant risk factor for stomach cancer and mucosa-associated lymphoid tissue (MALT) lymphoma developed in adults [1].

A published study on the pediatric population found that the prevalence rates of *H. pylori *infection range from 1.8% to 65% among children [2]. However, data on the prevalence of this infection in Morocco is limited to adults, such as the 69.4% detected by biopsy culture in Rabat [3]. The diagnosis of *H. pylori* infection is possible by invasive and non-invasive tests. Among the invasive methods, histology was the first method used to detect *H. pylori* providing additional and essential information on the status of the mucosa [4]. Much is known about the association of *H. pylori* infection with gastrointestinal symptoms in adults, such as nausea, vomiting, and abdominal pain [5], but its role in children is unclear. The clinical manifestations of this infection at this age are often modest, and nonspecific [6]. In addition to symptoms as clinical factors of *H. pylori* infection, factors such as age, gender, and a variety of socioeconomic indicators are associated with the prevalence of *H. pylori* infection [7].

In addition, another aspect of *H. pylori* infection is its histopathological characteristics. As a pathogen, *H. pylori* is highly adapted to the gastric mucosa, which causes gastritis in all infected individuals. Most pediatric studies report histological gastritis in all *H. pylori* infections. The proportion of patients with this inflammation ranges from 75% to 100% [8]. In addition to peptic ulcers and gastric cancers, *H. pylori* gastritis has been described by several studies, which reported the presence of *H. pylori* with the histological lesions of gastric mucosa in the pediatric population [9]. Accordingly, this study examined and described demographical, clinical, endoscopic, and histopathological aspects of *H. pylori* infection in Moroccan children. To the best of our knowledge, this is the first study of its kind in our context.

## Materials and methods

A cross-sectional study was conducted at the Hassan II University Hospital in Fez, Morocco, from January 2019 to January 2021. Outpatients (under 17 years of age) who required diagnostic or therapeutic upper gastrointestinal endoscopy and had not taken a drug within 30 days of the testing (antibiotics or proton pump inhibitors) were included randomly in this study. Informed consent and a structured questionnaire containing sociodemographic and clinical information were obtained from the patient's parents as approved by the Research Ethics Committee of the University Hospital of Fez, Morocco (approval number: 28/20).

The endoscopic lesions were recorded and biopsy specimens were captured during endoscopy supervised by a medical gastroenterologist with the support of trained nurses and auxiliary staff. Two biopsy specimens were collected, one at the antrum and one on the corpus. They were fixed in 10% formalin, embedded in paraffin, and cut at 6 mm. Histological sections were stained with hematoxylin and eosin (H&E) and modified Giemsa for light microscopy [10]. They were analyzed according to the revised Sydney System [11] by a pathologist who noted the presence or absence of *H. pylori* and the histopathological funding. The histological variables (topographic of gastritis inflammation, intensity of inflammation, gastritis activity, gastric atrophy, intestinal metaplasia, and intestinal dysplasia) were graded according to the updated Sydney System visual analog scale to generate a score (0 = absent, 1 = mild, 2 = moderate and 3 = severe). Subjects were considered infected when the *H. pylori* bacterium was detected in histological sections stained with Giemsa.

The mean age and the proportions of the categorical data were calculated using a simple descriptive analysis. Bivariate analysis was carried out using the chi-square (χ^2^) test and the binary linguistic for comparing and exploring the distribution of the categorical variables in the infected and no infected children groups. A p-value ≤ 0.05 was considered significant.

## Results

**Figure 1:**
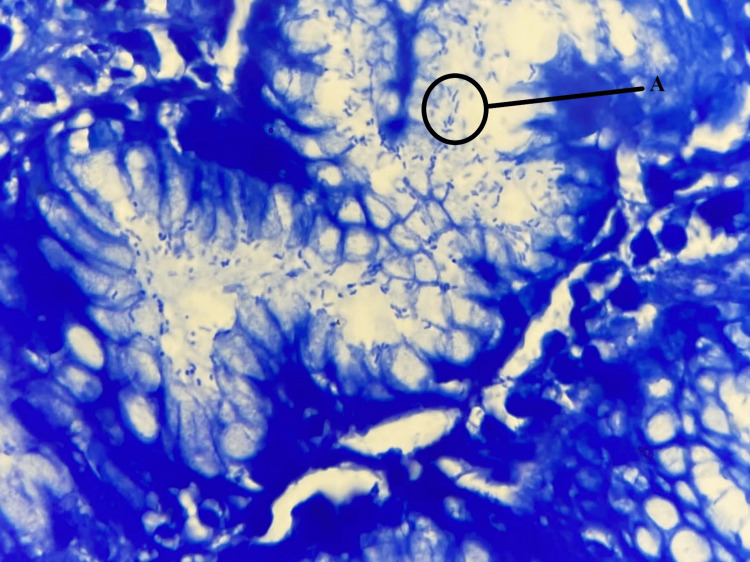
Helicobacter pylori in Giemsa staining A: *Helicobacter pylori *bacterium

In this study, 213 children with symptoms (age range 1-17 years) were included of which 116 (54%) were females and 97 (46%) were males. According to the results of the Giemsa stain (Figure 1), 95 children (45%) presented *H. pylori* infection and 118 (55%) did not (Table 1). Among several factors studied about *H. pylori* positivity, the factors tested in the present study were gender and age. Table 2 shows the frequency of *H. pylori* concerning gender and age group. There was no statistical difference in *H. pylori *frequency in gender (Table 2). Out of 97 males, *H. pylori* was positive in 47%, and of 116 females, it was positive in 44%. There was, however, a significant association between *H. pylori* infection and age (Table 2). The rate of a positive number increased with children’s age (Table 2) from 26% in the age group of one to four years to 65% in the older age groups (12-17 years). In the age group of 5-8 years and 9-12 years, we recorded rates of 49% and 52%, respectively.

**Table 1:** Helicobacter pylori infection according to hematoxylin and eosin and Giemsa staining

	Hematoxylin and Eosin		Giemsa	
	Frequency	Percent	Frequency	Percent
*Helicobacter pylori* status				
Negative	130	61%	118	55%
Positive	83	39%	95	45%

**Table 2:** Demographic characteristics of all enrolled patients

	Number (%)	Negative *Helicobacter pylori* N (%)	Positive *Helicobacter pylori *N (%)	p	OR	CI
Gender						
Female	116 (54)	65 (56%)	51 (44%)	0,513	1,198	0,697-2,058
Male	97 (46)	51 (53%)	46 (47%)			
Age group						
< 5 years	65 (30)	48 (74%)	17 (26%)	0,0001	-	-
5-8 years	59 (28)	30 (51%)	29 (49%)			
9-12 years	57 (27)	27 (48%)	30 (52%)			
> 12 years	32 (15)	11 (35%)	21 (65%)			

As shown in Table 3, vomiting in 79 cases (37%) followed by epigastric pain in 47 patients (22%) were the most common clinical indications for endoscopy. There was no significant relationship between *H. pylori* infection and all of the symptoms cited in this study. According to the gastric endoscopy findings (Table 4), *H. pylori* infection was associated only with the presence of nodular gastritis (p<0.05). 

**Table 3:** Clinical characteristics in all enrolled patients

	N (%)	*Helicobacter pylori* negative N (%)	*Helicobacter pylori* positive N (%)	Sig	OR	95%CI
Anemia	12 (5.63%)	9 (75%)	3 (25%)	0,079	0,275	0.065 - 1.159
Chronic diarrhea	13 (6.1%)	5 (38.46%)	8 (61.53%)	0.191	2.342	0.653 - 8.397
Work-up for celiiac disease	24 (11.26%)	12 (50%)	12 (50%)	0.938	1.039	0.401 - 2.685
Dysphagia	14 (6.57%)	7 (50%)	7 (50%)	0.641	1.320	0.411 - 4.235
Gastroesophageal reflux disease	5 (2.34%)	3 (60%	2 (40%)	0.771	0.756	0.115 - 4.941
Vomiting	79 (37.08%)	49 (62.02%)	30 (37.97%)	0.136	0.622	0.332 - 1.162
Epigastric pain	47 (22.06%)	25 (53.19%)	22 (46.80%)	0.747	1.123	0.554 - 2.277
Hematemesis	19 (8.92%)	8 (42.1%)	11 (57.89%)	0.151	2.202	0.749 - 6.467
Ingestion of a foreign object	6 (2.81%)	2 (33.33%)	4 (66.66%)	0.340	2.369	0.402 - 13.926
Suspected caustic ingestion	5 (2.34%)	2 (40%)	3 (60%)	0.574	1.701	0.267 - 10.813

**Table 4:** Endoscopic findings in all enrolled patients

	N (%)	*Helicobacter pylori* Negative, N (%)	*Helicobacter pylori* Positive, N (%)	Sig	Odds	95%CI
Melena	5 (2.34)	4 (80)	1 (20)	0.344	0.331	0.033 - 3.269
Gastric stenos	25 (11.73)	14 (56)	11 (44)	0.496	1.377	0.547 - 3.460
Erythematous gastritis	45 (21.12)	25 (56)	20 (44)	0.669	0.857	0.421 - 1.740
Nodular gastritis	65 (30.51)	23 (35)	42 (65)	0.000	3.146	1.679 - 5.891
Petechial gastritis	14 (6.57)	7 (50)	7 (50)	0.528	1.435	0.467 - 4.401
Esophagitis	35 (16.43)	24 (69)	11 (31)	0.063	0.454	0.197 - 1.044

On histopathological examination of 213 specimens (Table 5), the lesions of chronic inflammation observed in a majority of the infected children (98%) were atrophic in 91 cases (47%), active in 42 patients (22%), and with lymphoid follicles aspect in 56 cases (57%). Among the infected group, and according to the gastritis grading, the chronic inflammation intensity ranges from mild in 83% to moderate in 11% and severe in 4%; the activity of gastritis was mainly absent in 72% or mild in 26% and the atrophy varied from mild (48%) to moderate (5%).

**Table 5:** Histopathological features in all enrolled patients N/n: number of cases observed in the sample size/sample size

	N/n (%)	*Helicobacter pylori* Positive, N (%)	Sig	OR	95% CI
Histological characteristics					
Chronic gastritis	193/213 (91%)	96/98 (98%)	0.042	4.939	1.058 - 23.062
Atrophic gastritis	91/193 (47%)	52/98 (53%)	0.089	1.675	0.923 - 3.041
Active gastritis	42/193 (22%)	27/98 (28%)	0.156	1.699	0.816 - 3.537
Follicular gastritis	97/213 (45%)	56/98 (57%)	0.077	1.7	0.942- 3.068
Intestinal metaplasia	3/213 (1%)	0	0.999	0.000	-
Dysplasia	0	0			
Topographic of gastritis					
Antral	62/213 (29%)	28/98 (29%)	0.006	-	-
Fundic	44/213 (21%)	22/98 (22%)			
Pangastric	87/213 (41%)	46/98 (47%)			
Normal mucosa	20/213 (9%)	2/98 (2%)			
chronic inflammation					
No inflammation	20/213 (9%)	2 (2%)	0.001	-	-
Mild	168/213 (79%)	81/98 (83%)			
Moderate	21/213 (10%)	11/98 (11%)			
Severe	4/213 (2%)	4/98 (4%)			
Grade of atrophy					
No atrophy	122/213 (57%)	46/98 (47%)	0.023	-	-
Mild	80/213 (38%)	47/98 (48%)			
Moderate	10/213 (5%)	5/98 (5%)			
Severe	1/213 (0%)	0 (0%)			
Activity of gastritis					
No active	171/213 (80%)	71/98 (72%)	0.017	-	-
Mild	40/213 (19%)	25/98 (26%)			
Moderate	2/213 (1%)	2/98 (2%)			

## Discussion

*H. pylori* was detected in the present study by histological examination based on Giemsa staining. This is a method of choice, cheap, easy to perform, and reproducible. It is significantly more sensitive than other special stains as H&E used to appreciate the gastric mucosa [12].

We found that the prevalence of *H. pylori* infection in our study (45%) was similar to that in a study conducted on symptomatic Saudi children (49%) [13], and it was higher than 24.7%, and 14.6% reported respectively in a Brazilian pediatric and adolescent population [14], and in Jordan [15]. Thus, our result confirms a higher prevalence of *H. pylori* in the Moroccan population just like a study conducted in our area in a group of 429 adults (69.9%) [16]. The Rabat region reported a rate of 69.4% based on the results of a multicenter study on symptomatic adults [3].

The higher prevalence of *H. pylori* infection could be attributed not only to the sociodemographic, environmental, behavioral, and clinical factors but also to other factors that could significantly influence the variation in results among the studies, including inclusion criteria, sample size, method of diagnosis, and presence of the gastrointestinal symptoms, which provided a growth medium (changing pH, thinned gastric wall, gastric ulceration, and altered gut microbiota) for the bacteria, so the participants were more likely to contract *H. pylori* [17].

Among demographic factors, there are no differences in *H. pylori* infection rate in both girls and boys (p=0.513, OR = 1,198) in our study. Regarding age, similar to that reported by other authors [18], the prevalence of this infection increased with age and reported values of 26%, 49%, 52%, and 65% in the age groups of one to four years, five to eight years, 9-11 years, and 12-17 years, respectively. In addition, there was an increased rate of *H. pylori* infection in older children, which could be explained by person-to-person transmission of the bacterium. These factors are restricted to family members during neonatal and preschool years, reducing the risk of *H. pylor*i gastroenteritis in the youngest children compared with the older school-age children exposed to more sources of infection in an environment [19]. In this direction, it is reported that the low infection rate of preschool children (0.6%) was significantly increased in high school students (13.5%) (P =0.001) [19].

Regarding the clinical factors of *H. pylori* infection, the population evaluated in this study was characterized by symptoms represented mainly in celiac disease suspicion, dysphagia, gastroesophageal reflux disease, vomiting, and epigastric pain. The current study showed that the children’s symptoms distribution for *H. pylori*-positive cases was not statistically significant and similar to that reported by Altamimi et al. who reported no association between the majority of gastrointestinal symptoms and being infected with *H. pylori* [15]. In addition, according to some authors, the *H. pylori* infection was associated with epigastric pain. But it was mainly either clinically silent or associated with nonspecific signs or symptoms according to results of various childhood studies [20].

In endoscopy, among the infected group, the current study showed that nodular gastritis was the most common gastritis status related to *H. pylori* infection. It was seen in 42 cases (64.61%), higher than that reported by Luzza et al. (40%) [21]. Similar to our results, and according to several authors, nodular gastritis was the only which showed a significant statistical association with *H. pylori* infection and may be a pathognomonic macroscopic finding of childhood *H. pylori* infection [22]. In this respect, the positive predictive value of this endoscopic status in *H. pylori* infection ranged between 46% and 73% in the literature [23].

Histologically, our results demonstrated that chronic gastritis was mainly seen in the infected group (98%), similar to that found by Mazigh-Mrad et al. (88.5%) [24]. A similar rate (87.7%) was reported by Al Kirdy et al. in infected patients [25]. The same study showed that active gastritis was seen in most of the infected patients (84.9%). A value of 63% was reported by Mazigh Mrad et al. [24], higher than that shown in our study.

Regarding gastritis degree, chronic gastritis was commonly mild in 83% and moderate in 11% of the infected cases in our study. It was severe in only four infected children (4%). Similarly, Cárdenas-Mondragón et al. reported that *H. pylori* infection was usually associated with mild to moderate inflammation in children [26]. In addition, according to the analysis of these authors, the rarest cases of severe gastritis could be explained by coinfection *H. pylori* bacterium and Epstein Barr virus [26]. Regarding the activity of gastritis and similar to that registered by authors [26], It was mild in 26% to absent in 72% of the infected group in our population.

The rate of gastric atrophy in the infected group was higher (53%) than that reported by several authors in another context as Guiraldes et al. [8], and Campbell et al. [27] with values of 0 and 2/83. It was lower than that reported in a Turkish country (72%) characterized by a similar prevalence of *H. pylori* to our context (43.9-53%) [28]. In addition, gastric atrophy was not only observed in adult patients [29], it was estimated to vary from 0% to 72% in different studies in pediatric populations [30], which suggests the need for close and extended clinical and endoscopic surveillance of children with atrophic gastritis and identifying other factors (excluding *H. pylori*) that may predispose them to gastric atrophy [30].

The principal limitation of this study was the exclusion of eradication results and the therapeutic strategy used for managing our patients. It was not possible due to the study design and the inclusion of different diseases of the stomach. Thus, we considered the need for further research using an eradication test for* H. pylori *infection as a urea breath test and focusing on one of the stomach diseases individually.

## Conclusions

In the present study, we were able to characterize *H. Pylori* infection in Moroccan children, where this infectious disease was characterized by nodular gastritis and gastric atrophy, indicating that clinical, endoscopic, and histopathological findings should be combined for monitoring the mucosa of children.
